# The screening power of methylenetetrahydrofolate reductase C677T polymorphism versus plasma homocysteine concentration in patients with stenosis of the internal carotid artery

**DOI:** 10.1186/1477-9560-4-16

**Published:** 2006-09-25

**Authors:** Robert Loncar, Barbara T Müller, Reiner B Zotz, Christof Sucker, Wilhelm Sandmann, Rüdiger E Scharf

**Affiliations:** 1 Department of Hemostasis and Transfusion Medicine, Heinrich Heine University Medical Center Duesseldorf, Germany; 2 Department of Vascular Surgery and Kidney Transplantation, Heinrich Heine University Medical Center Duesseldorf, Germany

## Abstract

**Background:**

Hyperhomocysteinemia is an important and independent risk factor for vascular disease. About 35% of patients with stroke and 47% of patients with peripheral arterial disease have elevated plasma homocysteine (HCY) concentrations. The relationship between plasma HCY and the methylentetrahydrofolate reductase (MTHFR) C677T polymorphism is still unclear, especially in regard to screening/diagnostic power.

**Methods:**

This case-control study was performed on 96 patients, who underwent surgery due to asymptomatic or symptomatic high grade stenosis of the internal carotid artery (ICA), and 96 healthy age and sex-matched, controls. Plasma HCY concentration was determined using a commercial kit for fully automated analysis (AxSYM, Abbott). The C677T polymorphism of the MTHFR-gene was assessed by PCR.

**Results:**

The mean plasma HCY concentration was significantly higher in the group with stenosis of ICA compared to the controls, 12.43 ± 6.96 μM and 10.16 ± 3.16 μM, respectively, (p < 0.05). An HCY plasma concentration of 1.5 SD above the mean value of the control group, was defined as cut-off for a pathological versus physiological plasma concentration. The sensitivity and specificity of HCY was 0.27 and 0.94, respectively. The positive predictive value was 0.82. There was no significant difference in the frequency of the MTHFR 677 CT and TT genotype between patients and controls (47% vs. 47% and 8.3% vs. 11.4%, respectively). Carriers of the T-allele (CT and TT genotypes) have significantly higher plasma HCY concentrations than CC patients, 14.1 ± 7.6 μM and 10.29 ± 5.2 μM, respectively, p < 0.05. Sensitivity and specificity of the MTHFR C677T polymorphism (T-allele) were 0.56 and 0.40, respectively. The positive predictive value was 0.48. There was no significant difference in plasma HCY or genotype frequency of the MTHFR C677T polymorphism between asymptomatic and symptomatic patients.

**Conclusion:**

Our study shows that in a population with a given pretest disease probability of 50%, the determination of plasma HCY concentration, with a positive predictive value of 0.82, is more suitable for screening of patients at risk than analysis of the MTHFR C677T polymorphism.

## Background

Stroke is a frequent cause of death in the developed world with a prevalence of 1–5% in the general population. In Germany, there are about 160,000 new cases of stroke recorded per year, with an annual cost over 11.7 billion dollars [1,2]. About 15% of all strokes are caused by atherosclerotic stenosis of the carotid bulb and the ICA [3]. Recent studies have demonstrated a reduction of stroke by prophylactic surgical treatment of asymptomatic high grade ICA stenosis [4] as well as by surgery for symptomatic ICA stenosis in patients who have already experienced a transient ischemic attack (TIA) or a stroke [5]. As recommended by the stroke council of the American Heart Association the best approach to reduce the burden of stroke still remains its prevention. High-risk stroke individuals have therefore to be identified and targetted for specific interventions [1].

In 1969, McCully reported an autopsy case of a 7-week-old-new-born with advanced atherosclerotic lesions and very high plasma homocysteine concentration [6]. The author speculated that the arterial damage was associated with increased concentrations of HCY or a derivative of HCY. Elevated plasma HCY concentrations are observed in about 13% of patients with coronary artery disease, 35% of patients with stroke and up to 47% of patients with peripheral arterial disease [7-11]. Plasma HCY concentration may be significantly elevated as a result of interaction of genetic and acquired factors [12]. Two important observations have put the research on HCY in focus. First, hyperhomocysteinemia has been established as an important and independent risk factor for vascular disease. Thus, a 5 μM increase in plasma HCY concentration may be associated with a 25–30% increase in risk of vascular events [13]. Second, an elevated HCY concentration can be normalized by vitamin substitution (folic acid, pyridoxine hydrochloride (B6) and cyanocobalamin (B12) with consecutive deceleration of atherosclerotic processes [12-17].

With respect to genetic factors, homocysteinemia may be the result of enzyme deficiency of the homocysteine intermediary metabolism. Apart from rare inherited metabolic disorders (errors in cobalamin metabolism, cystathionine β-synthase deficiency), the most common disorder investigated has been a deficiency of methylentetrahydrofolate reductase (MTHFR, EC 1.5.1.20). Frosst et al. [18] identified a C-to-T substitution at nucleotide 677 that converts alanine to valine (MTHFR C677T polymorphism). The mutation of the heterozygous or homozygous form correlated with reduced enzyme activity and increased thermolability in lymphocyte extracts [18,19]. This is the most common mutation and has a prevalence in the general population, 5–10% as TT and up to 40% as CT genotype [20,21]. Nevertheless, due to the high incidence in the general population and its physiological role, the 677C-T mutation may represent an important genetic risk factor of homocysteine associated vascular disease [18,20,21]. Kang et al. suggested that thermolabile MTHFR was associated with the development of coronary artery disease [22]. A generally accepted hypothesis is that homozygous TT-genotype leads to the hyperhomocystinemia with a consecutive premature vascular disease. On the other side, heterozygous carriers have mild hyperhomocysteinemia with a predisposition for accelerated atherosclerosis [19,20,22]. Although determination of different polymorphisms became a standard diagnostic tool, the relationship between MTHFR C677T polymorphism, related clinical entities and plasma homocysteine concentration is still not fully understood [23-25].

We performed a case-control study in the patients who were admitted for surgical treatment of high grade ICA stenosis to assess the screening and/or diagnostic power of the MTHFR C677T polymorphism against plasma homocysteine concentration. This study was conducted in order to improve our knowledge on the role of the MTHFR C677T polymorphism and plasma HCY in patients with ICA stenosis, to rationalize the diagnostic work-up and to subsequently reduce unnecessary costs in routine diagnostics.

## Methods

The patient group consisted of 96 persons (age range 40–87 years) who were admitted to our hospital for surgery due to a high-grade carotid artery stenosis (at least 80%). Thirty-four patients were asymptomatic and 62 symptomatic and had already experienced TIA or stroke. The diagnosis of ICA stenosis was confirmed by doppler and duplex sonography in all patients and, additionally, by digital subtraction angiography (DAS) or magnetic resonance angiography (MRA) in some cases. All patients had a cranial computed tomography (CT) or magnetic resonance imaging (MRI) performed to evaluate preoperative brain damage. Ninety-six healthy volunteers (age range 45–83 years) without evidence of atherosclerosis or deep vein thrombosis represented the control group. The sex distribution was uniform in both groups, 23% (n = 22) women and 77% (n = 74) men, respectively.

A careful anamnestic evaluation and physical examination were performed in all participants including evaluation of nutrition. Patients with vitamin and mineral supplementation and subjects with known disorders of B6, B12 and folate metabolism, as well as patients suffering from hematological and oncological disorders were excluded. Furthermore, subjects under continuous or recent medication, which are known to influence HCY, folic acid and vitamins B metabolism, women with hormone contraception or any other form of hormone substitution were also excluded. This study was performed according to the Helsinki declaration and was approved by the local ethical committee. A written consent has been obtained from each participant.

### Biological sample collection

Venous blood was obtained by puncture of the cubital vein, collected in EDTA tubes and immediately centrifuged at 2500 rpm for 10 min at 4°C. Cells were further assessed for genetic polymorphism. The plasma fraction was aliquoted for biochemical analysis. One aliquot was stored at -40°C to await analysis of homocysteine, folic acid or B12.

### Homocysteine assay

Homocysteine plasma concentration was measured using a standard commercial diagnostic kit (AxSYM Homocystein) for the fully automated Abbott AxSYM system (Abbott Diagnostic Division, Wiesbaden, Germany). Briefly, the assay included 3 analytical steps: 1) reduction of protein-bound homocysteine and other homocysteine fractions to free homocysteine using dithiothreitol, 2) enzymatic conversion using synthetic activity of S-adensylhomocysteine (SAH)-hydrolase in excess of adenosine, 3) a fluorescence polarisation immunoassay (mouse anti-SAH antibody) for determination of S-adenosylhomocysteine. Linearity of homocysteine determinations in the water and human whole plasma were proven using serial dilutions of HCY standards (range 0.6–600 μM). Intra- and inter-assay variations and possible effects of freezing and thawing were also assessed. According to the results published by others and following the manufacturer's recommendations, the cut-off value for an elevated HCY concentration was set at 1.5 SD above the mean value of the control group.

### Determination of plasma folic acid and B12 concentration

Folic acid and vitamine B12 plasma concentrations were assessed in one aliquot of stored plasma using standard commercial Abbott kits, the AxSYM Folate Assay and the AxSYM B12 Assay. AxSYM Folate assay is a Ion-Capture-Assay for quantitative determination of folic acid in human sera or plasma and AxSYM B12 is a microparticle enzyme intrinsic factor assay (MEIA) for quantitative determination of vitamin B12 in human serum or plasma. Both assays were conducted according to the manufacturer's instruction using AxSYM analyzer.

### MTHFR C677T polymorphism

The MTHFR C677T polymorphism was assessed using the Lightcycler-MTHFR C677T mutation detection protocol, previously described in detail [26]. Briefly, the mutation detection protocol was adapted for high-speed PCR in glass capillaries using the Lightcycler Instrument (LightCycler^® ^Roche Diagnostics GmbH, Mannheim, Germany) and hybridisation probes for genotyping of the C677T point mutation in exon 4 of the MTHFR gene in human DNA. A 233 bp fragment of the MTHFR gene is amplified from genomic DNA using specific primers and the PCR product is detected by fluorescence (Lightcycler -Red 640) using a specific pair of hybridisation probes.

### Statistics

The data are presented as means ± SD. Statistical significance of the results was analysed using the Student's t-test. In cases where the data distribution was not compatible with the application of Student's t-test, Wilcoxon's test of the sum of ranks was applied. Regression analysis was based on individual measurements using the Spearman's rank correlation coefficient. In addition, non-linear regression analysis was compared with the rank correlation analysis. Statistical analyses were performed using SPSS for Windows, version 6.0.1. A P-value below 0.05 (two-tailed) was assumed to indicate a significant difference.

Within both, the patient and the control group, interindividual differences of the plasma HCY concentration were expressed as the coefficient of variation (CVobs). To obtain the real biological variability of homocysteine plasma concentration, the observed variability was corrected for methodological variability, which was assessed separately using plasma sample standards with known concentrations of homocysteine and expressed as the coefficient of variation of the method (CVmeth). A real biological coefficient of variation was calculated as CVbiol = (CVobs^2^-CVmeth^2^)^1/2^. For evaluation of the HCY plasma concentration and MTHFR C677T polymorphism, as a marker of enhanced risk of ICA stenosis, Bayes theorem was used [27].

## Results

Linearity of HCY plasma determination in human plasma was tested by adding aliquots of known HCY standards to plasma and processing the samples as described above. The average recovery was 104% and 107% in water and plasma dilutions, respectively. Correlation between diluted HCY standards in water and human plasma HCY measurements was 0.94. Temperature changes (freezing, thawing), which may affect homocysteine distribution among protein-bound, free and mixed disulphides had no influence on the accuracy of the assay. Intra- and inter-assay variations as well as the coefficients of variation with and without freezing, were identical, yielding a coefficient of variation of the method (CVmeth) of 0.05. Descriptive statistical data for total plasma HCY and MTHFR C677T polymorphism are summarized in the Table 1. There was a significant difference between the control and patient group regarding total plasma HCY (p < 0.05). The plasma HCY in the patients averaged 12.43 ± 6.96 μM (range 3.4 to 40.8 μM) and showed significantly higher biological coefficient of variation (CV) related to the control, 0.47 vs. 0.30 respectively, p < 0.05. Although the average B12 and folate plasma concentration did not differ significantly between both groups (p > 0.05), a biological coefficient of variation of B12 and folate was 2.4- and 2.7-fold higher in the patients compared to the controls.

**Table 1:** Age, biochemical data and MTHR C677T polymorphism in the controls and patients with ICA stenosis

Parameter	Control	ICA	p
Age, years	66.8 ± 9.9	68.9 ± 8.9	>0.05
Total homocysteine, μM	10.16 ± 3.16	12.43 ± 6.96	<0.001
B12, pg/ml	324 ± 67	376 ± 188	>0.05
Folate, ng/ml	6.73 ± 1.28	6.62 ± 3.52	>0.05
MTHFR CC, %	40	44	>0.05
MTHFR CT, %	48	47	>0.05
MTHFR TT, %	11.4	8.3	>0.05

In the control group, male subjects showed higher total plasma HCY concentrations than female subjects (10.8 ± 3.35 μM vs. 9.30 ± 2.03 μM p < 0.05). This gender-related significant difference in the total plasma HCY concentration was not observed in the group with stenosis of ICA. Male patients had no significantly different plasma HCY as compared to females (12.72 ± 7.39 μM vs. 11.45 ± 5.27 μM, p > 0.05). In the control group, HCY correlated significantly with age (r = 0.30, p < 0.05), while in the patient group no such a relationship with age was observed (r = 0.18, p > 0.05). However, in the control and the patient groups no significant correlation was found between the HCY concentration and age, when related to gender (p = 0.34 and p = 0.63, respectively). An interesting observation was found in regard to plasma HCY and folate concentration. In both groups, a significant inverse relation between folate and plasma HCY was observed (r = -0.32 and r = -0.22, p < 0.05, respectively). Between patients with low and high folate concentrations (defined as < of 40 % and % > 140% of the mean plasma folate concentration), a statistical difference in the plasma HCY concentration was observed, 14.04 ± 6.41 vs. 10.15 ± 4.21 μM, p < 0.01, respectively. HCY in both examined groups did not show significant correlation with plasma B12 concentration, r = 0.01.

The frequency distributions of the MTHFR C677T polymorphism are summarized in Table 1. The MTHFR CC genotype was present in 44% of patients and 40% of controls. Heterozygosity for MTHFR (CT genotype) was present in a similar frequency in both groups (48% vs. 47%, p > 0.05). MTHFR TT genotype was confirmed in eleven control subjects and in eight patients. The frequency of the TT genotype was slightly higher in the control group but the difference was not statistically significant, 11.4% and 8.3%, respectively, p > 0.05. There was no significant difference in the frequency of the MTHFR 677T polymorphism related to the gender, neither in control subjects nor in patients with ICA stenosis.

The relation between MTHFR C677T polymorphism and plasma HCY as well as plasma B12 and folate concentration in the patient group is presented in the Table 2. Patients carrying the T-allele (CT and TT genotype) had significantly higher plasma HCY concentrations than CC patients, 14.1 ± 7.6 μM vs. 10.29 ± 5.2 μM, respectively, p < 0.05. Interestingly, there was no statistically significant difference between folate and B12 concentration in regard to the MTHFR C677T polymorphism, p > 0.05. The coefficient of correlation between HCY and folate as well as HCY and B12, in regard to the MTHFR 677T polymorphism, was not statistically significant; between HCY and folate in the patients, carriers of T-allele was r = -0.24 and in the CC-patients r = -0.14, p > 0.05. The highest coefficient of correlation was observed between HCY and folate concentration at CT-patients, r = -0.28, p = 0.06.

**Table 2:** Descriptive statistical data related to the MTHFR C677T polymorphism and plasma HCY concentration in patients with stenosis of ICA.

	ICA stenosis
CC-HCY, μM	10.29 ± 5.25
CT-HCY, μM	14.32 ± 7.66
TT-HCY, μM	12.81 ± 8.17
CC-B12, pg/mL	381 ± 210
CT-B12, pg/mL	376 ± 185
TT-B12, pg/mL	412 ± 85
CC-Folate, mg/mL	7.82 ± 4.10
CT-Folate, mg/mL	6.15 ± 3.08
TT-Folate, mg/mL	7.91 ± 2.21

For further evaluation, the patient population was stratified according to the clinical severity of ICA stenosis (TIA or stroke). Compared to asymptomatic patients, symptomatic patients had higher HCY plasma concentrations, but the difference was not statistically significant (12.83 ± 6 μM and 11.54 ± 8 μM, respectively, p > 0.05). The frequency of MTHFR 677 CT or TT genotypes did not differ between asymptomatic and symptomatic patients (38% and 53%, 8.8% and 8%, respectively, p > 0.05).

To evaluate the total plasma HCY concentration and the MTHR polymorphism as a marker of ICA stenosis, Bayes theorem was used [16]. The cut-off value for pathological plasma HCY concentration was set to the mean + 1.5 SD of the plasma HCY concentration, measured in the control group, equivalent to 14.9 μM. Using this cut-off value, 27% of the patients with ICA stenosis had increased total plasma HCY concentration compared to the remaining patients, 21.70 ± 6.9 and 9.17 ± 2.82 μM, respectively, p < 0.001. Both sensitivity and specificity of plasma HCY concentration for the risk of ICA were 27% and 94%, respectively (Table 3.). When the cut-off value was set to the mean plasma HCY concentration + 1SD, sensitivity was slightly enhanced and specificity was slightly reduced. In our study population with a given pre-test disease probability of 50%, the positive predictive value of total plasma HCY concentration was 0.82, according to the Bayes theorem. At the same time, sensitivity and specificity of the MTHFR polymorphism (both CT and TT genotypes) was 0.56 and 0.40, respectively. The positive predictive value was 0.48. Considering a prevalence of ICA stenosis in the general population of approximately 3% [15], the calculated positive predictive value for HCY and MTHFR (CT and TT genotype) was 0.12. and 0.02, P < 0.01, respectively.

**Table 3:** Sensitivity, specificity and positive predictive value of HCY plasma determinationand MTHFR C677T-polymorphism in patients with ICA stenosis.

	Sensitivity	Specificity	Positive predictive value
HCY (cut-off 14.90 μM)*	0.27	0.94	0.82
MTHFR (CT and TT)	0.56	0.40	0.48

*Cut-off value = 1.5 SD above the mean value of the control group

According to the discrepancy between recruited subjects in regard to gender distribution (23% females and 77% males represent the gender structure of 96 consecutive patients admitted to our clinic with related symptoms) a separate statistical analysis was performed.

The analysis revealed that homocysteine plasma concentrations between male patients and male controls showed a significant statistical difference, 12.72 ± 7.34 μM and 10.8 ± 3.35 μM, p < 0.05, respectively. The sensitivity and specificity in the male subgroup compared to the whole collective were 0.26 and 0.27, 0.96 and 0.94, respectively. A positive predictive value, calculated in the male subgroup, was 0.86 compared to the 0.82 in the all subjects. Similarly, a separate analysis of the MTHFR 677T polymorphism in the male subgroup did not influence the results. Sensitivity and specificity were 0.54 and 0.38 and did not differ significantly compared to the whole group (0.56 and 0.40, respectively). The positive predictive value was 0.46 (0.48 in the whole group).

## Discussion

In this study we explored the association between the MTHFR C677T polymorphism and plasma HCY concentration in patients with ICA stenosis. The main aim was to evaluate the relevance of this genetic marker in comparison to plasma HCY and to assess the screening and/or diagnostic power of the MTHFR C677T polymorphism against plasma homocysteine concentration.

The study provided the following major results:

1. Homocysteine plasma concentration was significantly higher in the patient group compared to the control.

2. The genotype frequency of the MTHFR C677T polymorphism revealed no difference between the patient group and the group of healthy individuals.

3. Patients, carrying the T-allele had significantly higher plasma HCY concentrations than CC patients.

4. There was no statistically significant difference between symptomatic and asymptomatic patients in regard to plasma HCY concentration and genotype frequency of the MTHFR C677T polymorphism.

5. The determination of plasma HCY concentration with a positive predictive value of 0.82 was more suitable for screening and monitoring of patients at risk than analysis of the MTHFR C677T polymorphism (positive predictive value 0.48).

In order to evaluate total plasma HCY and MTHFR C677T polymorphism as marker of ICA stenosis, this study (index test) was performed according to "gold standard" procedures [27]. This requires that the status of patients is precisely defined (definitive procedure) and that pre-test probability of the disease is known. It is important to realize the homogeneity of the groups in respect to the selection parameters. Under these conditions, the calculated statistics show that assessment of plasma HCY is superior compared to MTHFR genotyping (Table 3). The positive predictive value, which is defined as probability of disease, if the test result is positive, has been calculated according to the Bayes theorem. The Bayes theorem combines three factors – sensitivity, specificity and disease prevalence, in the examined population. To calculate the prevalence, that is incidence of the disease in the examined population, is the most critical. In our model population, the prevalence was precisely defined by selecting 96 patients with diagnosed ICA stenosis and 96 healthy individuals as controls. Table 3 shows a positive predictive value for HCY of 0.82 in regard to the incidence of 50 %. Taking into consideration that age and gender distribution as well as environmental and behavioural factors were similar in both groups, a sensitivity of 0.27 with a specificity of 0.94 indicated that an altered HCY metabolism could be involved in about one third of all cases of ICA stenosis, with a total probability of 94%. If the findings on the model population are transferred to the general population, the much lower incidence of ICA stenosis, namely approximately 3%, has to be considered [1]. Since approximately 27% of subjects with ICA stenosis had elevated HCY and 94 % of healthy controls had a normal total plasma HCY concentration, the positive predictive value is 0.12 for the general population. From these results we concluded that measurement of total plasma HCY concentration should not serve as a screening test for ICA stenosis in the general population but rather as a screening test and/or monitoring marker in certain high-risk groups (e.g. individuals with higher familiar incidence of ICA stenosis or other forms of peripheral arterial obstructive disease).

As it is evident from Table 1, the total plasma HCY was significantly higher in the patient group compared to controls. As cut-off value was set 1.5 SD above the mean value of the control group, HCY plasma concentration was increased in 27% patients and ranged between 3.8 and 40 μM. In addition to the difference in the mean HCY concentration, the coefficient of variation (CVobsv) of plasma HCY differed significantly between patients and controls (0.47 and 0.30, respectively, p < 0.05). Taking into consideration that the method used for plasma HCY measurement exhibited variation (CVmeth) of 0.05, the largest fraction of the noted coefficient of variation could be attributed to true biological variation. A possible explanation for the higher plasma HCY and the higher CV of plasma HCY in the patients could not be be given through the difference in the genotype frequency of MTHFR 677T polymorphism. Namely, the genotype frequency of the MTHFR 677T polymorphism revealed no difference between patients and healthy individuals.

However, higher plasma HCY concentration and CV of plasma HCY in the group of patients could not be explained by the MTHFR C677T polymorphism. A careful data evaluation showed that there was no consistent relationship between genotype frequency, vitamin status and plasma HCY. An inconsistent relation between examined determinants might be explained by the presence of some other unidentified genetic linkage or unknown risk factors, which could have an impact on the development of hyperhomocysteinemia.

One possibility is that other mutations affect the MTHFR gene and have influence on the plasma HCY concentration. Van der Put et al. [28] for first the time reported the presence of an A1298C mutation of the MTHFR gene. Although three common MTHFR polymorphisms (C677T, C1298A, C1711T) have already been reported [29], only one polymorphism (C677T) has been investigated intensively as a risk factor for occlusive arterial vascular disease. The MTHFR A1298C polymorphism causes a glycine to alanine substitution in the MTHFR protein and has an allele frequency similar to that of MTHFR C677T [28-30]. The significance of this polymorphisms requires, however, further investigation.

Nevertheless, the application of Bayes theorem in our study design indicated that determination of MTHFR 677T polymorphism as screening or diagnostic tool is of minor importance.

## Conclusion

Our study showed that in a population with a given pretest disease probability of 50%, the determination of plasma HCY concentration, with a positive predictive value of 0.82, is more suitable for screening and monitoring of patients at risk than an analysis of the MTHFR C677T polymorphism.

From our results we concluded that the measurement of the total plasma HCY concentration does not serve as a screening test for ICA stenosis in the general population but rather as a screening test and/or monitoring marker in certain risk groups (e.g. individuals with higher familiar incidence of ICA stenosis or other forms of peripheral arterial obstructive disease).

## Competing interests

The author(s) declare that they have no competing interests.

## Authors' contributions

RL: initiated the study, designed, coordinated and drafted the manuscript.

BTM: participated in the design of the study and recruited patients.

RBZ: performed the statistical analysis and helped to draft the manuscript.

CS: recruited patients and performed the measurement.

WS: participated in the design of the study.

RES: participated in the design and coordination of the study.
